# Supplementary material for: Physical Activity Level and Perspectives of Participants Transitioning from Onsite to Virtual Cardiac Rehabilitation during the Early COVID-19 Pandemic: A Mixed-Method Study
Source: J Clin Med. 2022 Aug 18;11(16):4838. doi: 10.3390/jcm11164838 (PMC9409687; doi:10.3390/jcm11164838)
Supplement: Supplementary file 1 [file jcm-11-04838-s001.zip › jcm-1848675-supplementary.pdf]

Table S1: Script with questions utilized to guide the Focus Group sessions

**Introduction**	1. Cardiac rehabilitation services are intended to provide patients with a program that will facilitate their recovery both physically and emotionally. Please tell us about your experience with CR.<br>• Probe: How long were you enrolled in the program?<br>• Probe: How did you feel your program of CR was progressing?
**Transition**	2. How did feel during the transition from in-person to virtual rehab during COVID-19?<br>• Probe: Safety concerns? Fears? Anxious?<br>• Probe: Has your health affected your exercise experience?<br>• Probe: What are you hearing about exercise during COVID-19?
**Key**	3. Let's talk about what it has been like to participate in virtual rehab. How regularly were you able to perform your exercise prescription at home?<br>• Probe: How did the virtual model compare to in-person participation?<br>• Probe: What have been the benefits? The concerns?
**Key**	4. Let's talk about barriers and facilitators that you have identified for exercising at home.<br>• Probe: What barriers have you identified?<br>• Probe: What prevents you from participating in physical activity?<br>• Probe: What facilitators have you identified?<br>• Probe: What motivates you to participate in physical activity?
**Key**	5. Let's talk about the influence of the rehabilitation program.<br>• Probe: What contributes/contributed to your decision to exercise at TRI and to continue in the virtual CR program they are providing?<br>• Probe: How much is/has CR contributing/contributed to your exercise maintenance during COVID-19?<br>• Probe: How has contact with a case manager and/or use of cardiac college influenced your activity?<br>• Probe: Was the experience what you expected from a rehab program?
**Ending**	6. What advice would you give to program providers to improve the overall quality of your experience?<br>7. What would help facilitate long-term maintenance following the program?

Conclusion

As we conclude our time together, we want to make sure that we have noted all the points that are important to you. __________will read a short summary of the major points raised in the discussion. Please let us know if there is something we missed or a point you would like to add or elaborate on. (member checking)

Thank you for taking the time to attend this focus group today and for sharing your views. We will be looking at what you and others have told us and will write out recommendations that we hope will be used by the services that you have been using, and to incorporate new ideas into virtual care in order to optimize cardiac care. If you have any questions or concerns after today, feel free to call _________at the number provided in your package. We will be happy to talk with you and answer any questions.
